# Synthesis and Antifungal Potential of Some Novel Benzimidazole-1,3,4-Oxadiazole Compounds

**DOI:** 10.3390/molecules24010191

**Published:** 2019-01-06

**Authors:** Ahmet Çağrı Karaburun, Betül Kaya Çavuşoğlu, Ulviye Acar Çevik, Derya Osmaniye, Begüm Nurpelin Sağlık, Serkan Levent, Yusuf Özkay, Özlem Atlı, Ali Savaş Koparal, Zafer Asım Kaplancıklı

**Affiliations:** 1 Department of Pharmaceutical Chemistry, Faculty of Pharmacy, Anadolu University, Eskişehir 26470, Turkey; ackarabu@anadolu.edu.tr (A.Ç.K.); betulkaya@anadolu.edu.tr (B.K.Ç.); uacar@anadolu.edu.tr (U.A.Ç.); dosmaniye@anadolu.edu.tr (D.O.); bnsaglik@anadolu.edu.tr (B.N.S.); serkanlevent@anadolu.edu.tr (S.L.); yozkay@anadolu.edu.tr (Y.Ö.); 2 Doping and Narcotic Compounds Analysis Laboratory, Faculty of Pharmacy, Anadolu University, Eskişehir 26470, Turkey; 3 Department of Pharmaceutical Toxicology, Faculty of Pharmacy, Anadolu University, Eskişehir 26470, Turkey; oatli@anadolu.edu.tr; 4 Open Education Faculty, Anadolu University, Eskişehir 26470, Turkey; askopara@anadolu.edu.tr

**Keywords:** benzimidazole, 1,3,4-oxadiazole, antifungal activity, ergosterol biosynthesis, cytotoxicity, molecular docking

## Abstract

Discovery of novel anticandidal agents with clarified mechanisms of action, could be a rationalist approach against diverse pathogenic fungal strains due to the rise of resistance to existing drugs. In support to this hypothesis, in this paper, a series of benzimidazole-oxadiazole compounds were synthesized and subjected to antifungal activity evaluation. In vitro activity assays indicated that some of the compounds exhibited moderate to potent antifungal activities against tested *Candida* species when compared positive control amphotericin B and ketoconazole. The most active compounds **4h** and **4p** were evaluated in terms of inhibitory activity upon ergosterol biosynthesis by an LC-MS-MS method and it was determined that they inhibited ergosterol synthesis concentration dependently. Docking studies examining interactions between most active compounds and lanosterol 14-α-demethylase also supported the in vitro results.

## 1. Introduction

The treatment of fungal infections continues to be a thought-provoking problem worldwide. Such infections vary in severity from superficial to complex fungal infections and most commonly affect immunocompromised patients [1]. In the past the affected patients have used numerous antibiotics and synthetic drugs, mostly without precautions, which has led to antifungal resistance to prescribed agents, therefore, the high morbidity and mortality caused by fungi are still increasing serious threat [2]. Since current antifungals do not meet the growing requirements of managing with life threatening infections, medicinal chemists are highlighting the need for the search of novel antifungal drugs.

Nucleic acids, protein, sterols and cell wall are known targets of the current agents in antifungal therapy [3]. The introduction of polyene antifungals, such as amphotericin B, became a milestone in clinic, however, infusional toxicity, nephrotoxicity and electrolyte imbalances limited its usage [4,5]. Several decades later, azoles were discovered as a result of the continued search for new and less toxic antifungals. Ketoconazole, the first presented compound in the early 1980s for the oral treatment of systemic fungal infections [6]. Azoles block the 14-α-demethylation of lanosterol into ergosterol, which is a major component of fungal cytoplasmic membranes and a bioregulator of membrane asymmetry, fluidity and integrity [3]. This blocking occurs via the coordination of the nucleophilic nitrogen of the azole heterocyclic ring as the sixth ligand of the heme ferric ion in the active side of lanosterol 14-α-demethylase (CYP51) and the interaction of the azole drug side chains with the CYP51 polypeptide structure [7,8]. Catecholase is another specific enzyme type in fungi and it is also responsible for biologically essential functions. Thus, inhibition of catecholase may terminate the vital functions of the fungi [9,10,11]. In addition to inhibition of such vital fungal enzymes, some strategies including inhibition of candida biofilm formation [12,13] and treatment with organoselenium based agents [14,15,16,17] are recent antifungal treatment options.

In spite of the widespread use of these agents, the development of new azole antifungal drugs has been constantly required in the clinical therapy due to a number of clinically important limitations such as drug-resistant strains, serious side effects, narrow spectrum and toxicity [18,19]. The improvement of new hybrid molecules, which can be used either alone or as part of a combination therapy, is generally considered as a functional strategy to cope with the growing problem of antifungal resistance. The combination of two or more biologically important azole scaffolds could generate a new class of azole based antifungal agents. To this end, we designed some hybrid compounds bearing benzimidazole and 1,3,4-oxadiazole rings based upon the studies that reported the antifungal potentials of such compounds [20,21,22,23]. The electron rich nature of azoles provides easily to interact with various enzymes and receptors via noncovalent interactions. Benzimidazoles are an indispensable class of heterocyclic family and promising candidates for developing biologically active compounds due to their various pharmacological activities [24,25,26,27,28,29,30,31,32,33]. Comprehensive biochemical and pharmacological studies reported their large potentiality to inhibit the growth of fungal strains [34,35,36,37,38]. Particularly, in some studies 5-fluoro or 5-chloro substituted benzimidazoles displayed significant antifungal activity [39,40,41,42,43]. 1,3,4-Oxadiazole nucleus is also a fertile source of bioactivity in drug discovery because of its varied biological activities [44,45,46,47,48,49,50]. Recently, our research group has been actively involved in developing novel antifungal agents through modifying various azole compounds. Previously, we reported novel benzimidazole-triazole and benzimidazole-thiazole hybrid compounds some of which possessed significant ergosterol biosynthesis inhibition at 0.78 µg/mL and 1.56 µg/mL concentrations, respectively [8,51,52]. Taking the above points in consideration and in continuation of our studies, in current research, we clubbed benzimidazole and 1,3,4-oxadiazole rings to optimize the overall structure for better and promising antifungal efficacy and to evaluate the effect of the 1,3,4-oxadiazole ring on activity.

## 2. Results and Discussion

### 2.1. Chemistry

**Scheme 1:**
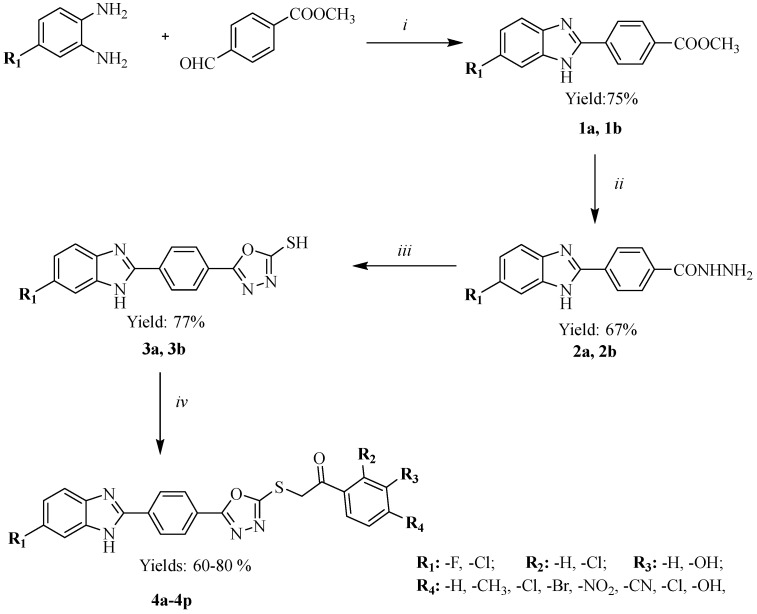
The synthetic protocol of the compounds. Reagents and conditions: (*i*) Na_2_S_2_O_5_, DMF, MWI, 10 min, (*ii*) NH_2_NH_2_xH_2_O, EtOH, MWI, 10 min, (*iii*) CS_2_/NaOH, EtOH, reflux, 8 h, (*iv*) appropriate phenacyl bromides, K_2_CO_3_, acetone, rt, 8 h.

The reaction sequences followed for the synthesis of the final compounds **4a**–**4n** are outlined in Scheme 1. Compounds **1** and **2** were synthesized according to reported methods [37]. The ring closure reaction of the corresponding hydrazide **2** with carbon disulfide in the presence of sodium hydroxide resulted in the formation of 5-(4-(1*H*-benzimidazol-2-yl)phenyl)-1,3,4-oxadiazole-2-thiol (**3**), which was then reacted with suitable substituted phenacyl bromides to obtain the target compounds **4a**–**4p**. The chemical structures of the compounds were elucidated via IR, ^1^H-NMR, ^13^C-NMR and HRMS spectroscopic methods. In the IR spectra of all compounds, the occurrence of N-H bonds were confirmed through bands in the region of 3325–3130 cm^−1^. The C=O bonds were confirmed through bands in the region of 1683–1658 cm^−1^.The characteristic C=N and C=C stretching vibrations were detected in between 1610–1462 cm^−1^. ^1^H-NMR spectra of compounds **4a**–**4p** are presented in the Supplementary Materials (Figures S1–S16). CH_2_-protons attached to carbonyl group resonated as singlet at 5.05–5.27 ppm in the ^1^H-NMR spectra of compounds. The singlet NH signal of benzimidazole was appeared at over 13 ppm in compounds **4a**–**4p**. The signals belonging to aromatic region was seen at 6.88–8.40 ppm. In the ^13^C-NMR spectra, the methylene carbons between the sulphur atom and carbonyl group was observed around 41 ppm just over the DMSO peaks. Two carbons on the oxadiazole ring were 163–165 ppm, while the carbonyl carbon was over 191 ppm. All other aromatic carbons were recorded between 96 ppm and 160 ppm. Also, carbon-fluorine coupling constants were observed for the first eight fluorinated derivatives. The HRMS spectra of compounds were found to be in full agreement with their molecular formula.

### 2.2. Biological Activity Screening

#### 2.2.1. Antifungal Activity

The in vitro antifungal activities of all synthesized derivatives **4a**–**4p** were screened at between 1 mg/mL–1.95 µg/mL concentrations using various *Candida* strains including *C. albicans* (ATCC 90030), *C. krusei* (ATCC 6258) and *C. parapsilopsis* (ATCC 22019) following the protocol of the EUCAST [53]. Amphotericin B and ketoconazole were selected as positive controls. The results of antifungal potential of the compounds presented in Table 1 indicated that among all strains, *C. albicans* was the most susceptible to compounds. Compounds **4h** and **4p** showed comparable activity with reference drugs. Compounds **4h** and **4p** were determined to have MIC_50_ values of 1.95 µg/mL against *C. albicans*, while that of amphotericin B was 1.95 µg/mL and ketoconazole was 7.8 µg/mL, respectively. Compound **4p** was the most potent derivative of the series, with MIC_50_ values of 1.95 µg/mL, 7.8 µg/mL and 31.25 µg/mL against *C. albicans*, *C. krusei* and *C. parapsilopsis*, respectively. Ketoconazole showed anticandidal activity with MIC_50_ values 7.8 µg/mL, 1.95 µg/mL and 1.95 µg/mL against *C. albicans*, *C. krusei* and *C. parapsilopsis*, respectively. The MIC_50_ values of amphotericin B were 1.95 µg/mL towards all *Candida* strains.

The synthesized compounds can be categorised as two classed based upon the presence of fluoro (compounds **4a**–**4h**) or chloro (compounds **4i**–**4p**) substituents at the C-5 position of the benzimidazole ring to investigate the structure-activity relationship of the compounds. The other factor providing the diversity of the compounds is the phenyl ring attached to the carbonyl moiety that carries different substituents at the C-2, C-3 or C-4 positions. The presence of a chloro substituent on the benzimidazole ring (compounds **4i**–**4p**) is generally more beneficial than a fluoro substituent (compounds **4a**–**4h**). In both analogues **4a**–**4h** and **4i**–**4p**, a bromo substituent at the C-4 position and a hydroxy substituent at the C-3 and C-4 positions of the phenyl ring enhance the activity. Compounds **4h** and **4p** in which two hydroxy groups are involved, forming a catechol moiety, proved to be the most active antifungal agent in the series. Catechol hydroxy groups, which are hydrogen bonding donors, are thus beneficial for high antifungal activity.

#### 2.2.2. Cytotoxicity Test

High selectivity for fungal cells compared to mammalian cells is an essential parameter in the development of novel antifungal agents. Therefore, we evaluated the cytotoxic effects of the most active compounds **4h** and **4p** on NIH/3T3 cells. The results of this investigation show that tested compounds are nontoxic at their active concentrations against NIH/3T3 cells (IC_50_ ≥ 500 ± 7.09 µg/mL and 141.291 ± 10.48 µg/mL, respectively). These results provide support for the idea that compounds **4h** and **4p** came to the forefront on the search of a new and safe antifungal candidate.

#### 2.2.3. Quantification of Ergosterol Level

The most widely used antifungal drugs (ketoconazole, fluconazole etc.) target CYP51 and inhibit ergosterol biosynthesis in fungal membrane. Based on this, ergosterol quantification was carried out to gain insight into the antifungal mechanism of action of the most active compounds **4h** and **4p**. The IC_50_ of these compounds and ketoconazole on ergosterol biosynthesis is outlined in Table 2.

A dose dependent decrease in ergosterol biosynthesis was detected with the treatment of tested compounds. This result reveals that the anticandidal effect of the tested compounds may arise from the inhibitory potential on ergosterol biosynthesis in a manner similar to that of the ketoconazole.

#### 2.2.4. Molecular Docking Studies

According to antifungal activity, compounds **4h** and **4p** were found as the most potent derivatives in the series and their ergosterol inhibition potency was proven via ergosterol quantification assay. In order to evaluate in silico features of these compounds, docking studies were performed on 14 α-sterol demethylase, which is a key enzyme for ergosterol biosynthesis in fungi.

As mentioned in anticandidal activity results, compounds **4h** and **4p** were the most effective especially on *C. albicans* with MIC_50_ value of 1.95 µg/mL. Thus, X-ray crystal structure originating from *C. albicans* (PDB ID: 5FSA) [54] was retrieved from the Protein Data Bank server (www.pdb.org) and docking studies were performed by using this crystal structure.

**Figure 1:**
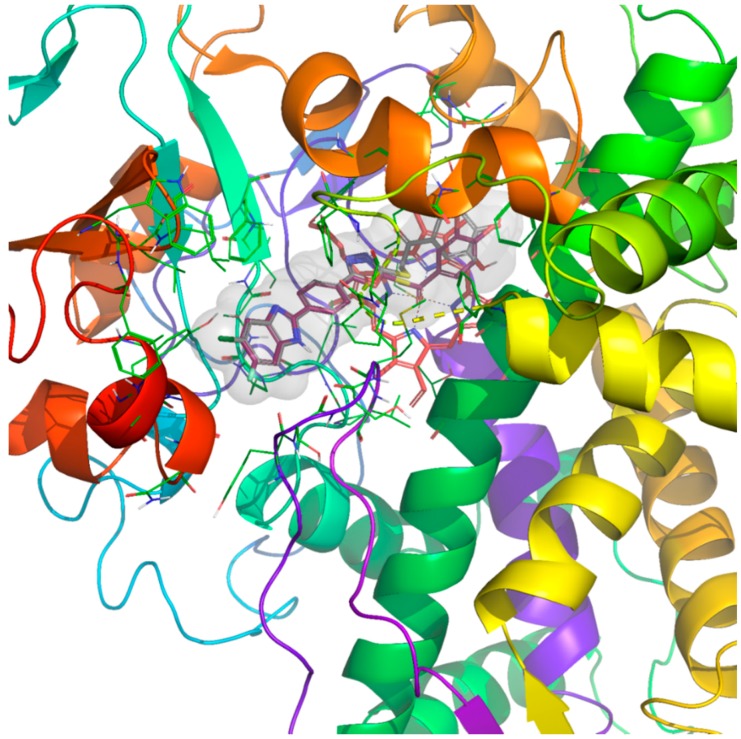
The superimpositions pose of active compounds **4h** and **4p** in the active region of 14 α-sterol demethylase (PDB code: 5FSA). The inhibitors **4h** and **4p** are colored with maroon and grey, respectively, and HEM with red.

**Figure 2:**
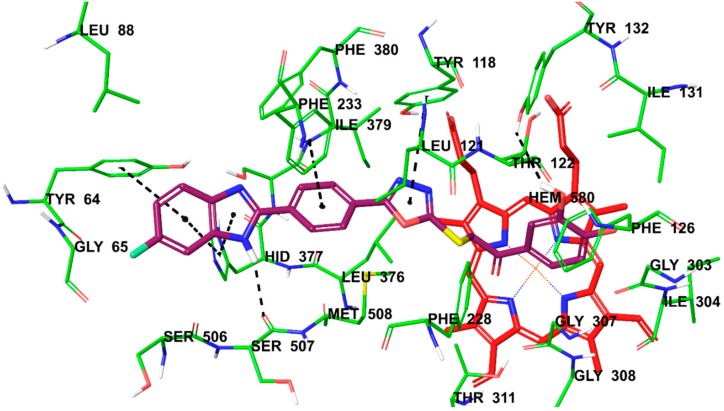
The interacting mode of compound **4h** in the active region of 14 α-sterol demethylase (PDB code: 5FSA). The inhibitor is colored with maroon and HEM with red.

**Figure 3:**
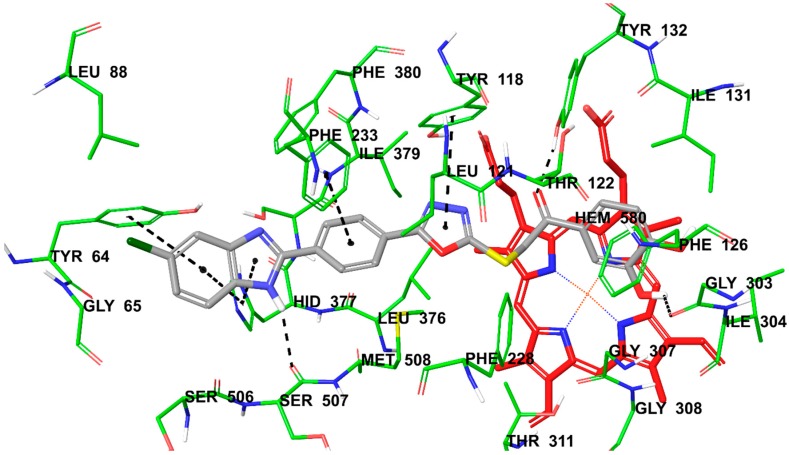
The interacting mode of compound **4p** in the active region of 14 α-sterol demethylase (PDB code: 5FSA). The inhibitor is colored with grey and HEM with red.

Figure 1, Figure 2 and Figure 3 present the docking poses of compounds **4h** and **4p**. It can be seen in these figures that compounds **4h** and **4p** show six common interactions. The fluoro or chloro substituted benzimidazole ring is especially important for hydrophobic binding profile. The benzo side of benzimidazole is in interaction with phenyl of Tyr64 by doing π–π interaction. Also, benzo and imidazole parts of benzimidazole create another π–π interaction with imidazole of Hid377 together. The NH moiety of benzimidazole is essential for polar interaction by forming a hydrogen bond with carbonyl of Ser507. Another common interaction for compounds **4h** and **4p** is related to phenyl between benzimidazole and oxadiazole rings in their structures. This phenyl establishes a π–π interaction with phenyl of Phe233. Furthermore, the last common interaction is another π–π interaction, which forms between oxadiazole ring and phenyl of Tyr118. The carbonyl moiety in compound **4p** forms a hydrogen bond with hydroxyl of Tyr132.

The only structural difference between compounds **4h** and **4p** is the presence of fluoro or chloro substitutions on benzimidazole ring, respectively. These compounds have 3,4-dihydroxyphenyl moiety unlike the other compounds in the series. The efficacy of these compounds is thought to be due in particular to hydroxyl functional groups. This idea has been proven by docking studies. According to Figure 2 and Figure 3, hydroxyl substituent at 3rd position of phenyl ring is in an interaction with hydroxyl of Tyr132 and carbonyl of Gly303 for compounds 4h and 4p, respectively. This means that 3rd position of phenyl ring may be important for inhibition of 14 α-sterol demethylase and ergosterol biosynthesis. Furthermore, the substituents capable of forming hydrogen bond such as hydroxyl moiety at this this position may be suggested for strong binding in the enzyme site. All these in silico findings help to explain the potent antifungal activity and ergosterol inhibition profiles of compounds **4h** and **4p**.

## 3. Materials and Methods

### 3.1. General Information

All chemicals employed in the synthetic procedures were purchased from Sigma-Aldrich (Sigma-Aldrich Corp., St. Louis, MO, USA) or Merck (Merck KGaA, Darmstadt, Germany). Melting points of the obtained compounds were determined by an MP90 digital melting point apparatus (Mettler Toledo, Columbus, OH, USA) and were uncorrected. ^1^H-NMR and ^13^C-NMR spectra of the synthesized compounds were registered by a Bruker 300 MHz and 75 MHz digital FT-NMR spectrometer (Bruker Bioscience, Billerica, MA, USA) in DMSO-*d*_6_, respectively. Splitting patterns were designated as follows: s: singlet; d: doublet; t: triplet; m: multiplet in the NMR spectra. Coupling constants (*J*) were reported as Hertz. The IR spectra were obtained on a Shimadzu, IR Prestige-21 (Shimadzu, Tokyo, Japan). M + 1 peaks were determined by Shimadzu LC/MS ITTOF system. All reactions were monitored by thin-layer chromatography (TLC) using Silica Gel 60 F254 TLC plates (Merck KGaA).

### 3.2. Chemistry

#### 3.2.1. Synthesis of methyl 4-(5(6)-substituted-1*H*-benzimidazol-2-yl)benzoates **1a**, **1b** and 4-(5(6)-substituted-1*H*-benzimidazol-2-yl)benzohydrazides **2a**, **2b**

The starting compounds **1a**, **1b**, **2a** and **2b** were synthesized in accordance to the method described in the literature [51].

#### 3.2.2. Synthesis of 5-[4-(5(6)-substituted-1*H*-benzimidazol-2-yl)phenyl]-1,3,4-oxadiazole-2-thiol derivatives **3a**, **3b**

Compounds **2a** or **2b** (0.031 mol) were dissolved in a solution of NaOH (1.48 g, 0.037 mol) in ethanol (150 mL). After addition of carbon disulfide (2.24 mL, 0.037 mol), the mixture was refluxed for 8 h. After this period, the solution was cooled and acidified to pH 4–5 with concentrated hydrochloric acid solution. The obtained solid was filtered, washed with water, dried and recrystallized from ethanol.

#### 3.2.3. Synthesis of 2-[(5-(4-(5(6)-substituted-1*H*-benzimidazol-2-yl)phenyl)-1,3,4-oxadiazol-2-yl)thio]-1-(4-substitutedphenyl)ethan-1-ones **4a**–**4n**

Compounds **3a** or **3b** (2 mmol) in acetone (40 mL) were stirred with an appropriate 4-substituted phenacyl bromide (2 mmol) and potassium carbonate (0.33 g, 2.4 mmol) at room temperature for 8 h. After the evaporation of acetone under reduced pressure, the product was washed with water, dried and crystallized from ethanol (96%).

*2-[(5-(4-(5(6)-Fluoro-1H-benzimidazol-2-yl)phenyl)-1,3,4-oxadiazol-2-yl)thio]-1-phenyl-ethan-1-one* (**4a**), Yield 71%, m.p. 262.4–265.7 °C. IR ν_max_ (cm^−1^): 3205 (N-H), 3053 (aromatic C-H), 2960 (aliphatic C-H), 1674 (C=O), 1597–1469 (C=N, C=C), 1313–1001 (C-N, C-O). ^1^H-NMR (DMSO-*d*_6_, ppm) δ 5.22 (2H, s, CO-CH_2_), 7.05–7.15 (1H, m, aromatic-H), 7.34–7.76 (5H, m, aromatic-H), 8.08–8.13 (4H, m, aromatic-H), 8.34 (2H, d, *J* = 8.4 Hz, aromatic-H), 13.26 (1H, s, NH). ^13^C-NMR (DMSO-*d*_6_, ppm) δ 41.1, 98.4 (d, *J* = 29.6 Hz), 105.0 (d, *J* = 22.3 Hz), 111.6, 112.8 (d, *J* = 9.5 Hz), 120.7, 124.3, 127.5, 127.6, 127.7, 129.0, 129.4, 132.3 (d, *J* = 13.6 Hz), 134.5, 135.5, 153.6 (d, *J* = 225.9 Hz), 164.1, 165.2, 193.2. HRMS (*m*/*z*): [M + H]^+^ calcd for C_23_H_15_FN_4_O_2_S: 431.0973; found: 431.0954.

*2-[(5-(4-(5(6)-Fluoro-1H-benzimidazol-2-yl)phenyl)-1,3,4-oxadiazol-2-yl)thio]-1-(4-methylphenyl)ethan-1-one* (**4b**), Yield 71%, m.p. 240.1–242.7 °C. IR ν_max_ (cm^−1^): 3305 (N-H), 3074 (aromatic C-H), 2995 (aliphatic C-H), 1672 (C=O), 1604–1498 (C=N, C=C), 1309–1031 (C-N, C-O). ^1^H-NMR (DMSO-*d*_6_, ppm) δ 2.42 (3H, s, CH_3_), 5.18 (2H, s, CO-CH_2_), 7.05–7.16 (1H, m, aromatic-H), 7.41 (2H, d, *J* = 8.0 Hz, aromatic-H), 7.48–7.59 (1H, m, aromatic-H), 7.69–7.73 (1H, m, aromatic-H), 7.99 (2H, d, *J* = 7.9 Hz, aromatic-H), 8.11–8.14 (2H, m, aromatic-H), 8.32–8.36 (2H, m, aromatic-H), 13.27 (1H, s, NH). ^13^C-NMR (DMSO-*d*_6_, ppm) δ 21.7, 41.0, 98.3 (d, *J* = 24.2 Hz), 105.0 (d, *J* = 24.7 Hz), 111.9, 112.7 (d, *J* = 8.0 Hz), 112.9, 120.7, 124.4, 127.5, 127.6, 129.1, 129.9, 131.2, 133.1 (d, *J* = 12.1 Hz), 145.1 (d, *J* = 3.0 Hz), 153.8 (d, *J* = 253.5 Hz), 164.2, 165.2, 192.6. HRMS (*m*/*z*): [M + H]^+^ calcd for C_24_H_17_FN_4_O_2_S: 445.1129; found: 445.1111.

*2-[(5-(4-(5(6)-Fluoro-1H-benzimidazol-2-yl)phenyl)-1,3,4-oxadiazol-2-yl)thio]-1-(4-chlorophenyl) ethan-1-one* (**4c**), Yield 67%, m.p. 253.6–255.3 °C. IR ν_max_ (cm^−1^): 3273 (N-H), 3088 (aromatic C-H), 2954 (aliphatic C-H), 1672 (C=O), 1589–1473 (C=N, C=C), 1292–1091 (C-N, C-O). ^1^H-NMR (DMSO-*d*_6_, ppm) δ 5.20 (2H, s, CO-CH_2_), 7.07–7.14 (1H, m, aromatic-H), 7.43 (1H, br s, aromatic-H), 7.56–7.62 (1H, m, aromatic-H), 7.68 (2H, d, *J* = 8.6 Hz, aromatic-H), 8.08–8.13 (4H, m, aromatic-H), 8.34 (2H, d, *J* = 8.5 Hz, aromatic-H), 13.26 (1H, s, NH). ^13^C-NMR (DMSO-*d*_6_, ppm) δ 41.0, 98.7 (d, *J* = 23.8 Hz), 105.1 (d, *J* = 25.3 Hz), 111.9 (d, *J* = 9.6 Hz), 124.3, 127.5, 127.7, 129.3, 129.5, 130.9, 132.3 (d, *J* = 12.6 Hz), 133.3, 134.2, 139.4, 141.5, 155.9 (d, *J* = 268.9 Hz), 164.0, 165.3, 192.3. HRMS (*m*/*z*): [M + H]^+^ calcd for C_23_H_14_ClFN_4_O_2_S: 465.0583; found: 465.0561.

*2-[(5-(4-(5(6)-Fluoro-1H-benzimidazol-2-yl)phenyl)-1,3,4-oxadiazol-2-yl)thio]-1-(4-bromophenyl)ethan-1-one* (**4d**), Yield 60%, m.p. 245.4–247.9 °C. IR ν_max_ (cm^−1^): 3261 (N-H), 3078 (aromatic C-H), 2937 (aliphatic C-H), 1681 (C=O), 1600–1473 (C=N, C=C), 1290–1076 (C-N, C-O). ^1^H-NMR (DMSO-*d*_6_, ppm) δ 5.27 (2H, s, CO-CH_2_), 7.06–7.13 (1H, m, aromatic-H), 7.42 (1H, br s, aromatic-H), 7.62 (1H, br s, aromatic-H), 8.12 (2H, d, *J* = 8.5 Hz, aromatic-H), 8.32 (4H, t, *J* = 8.6 Hz, aromatic-H), 8.40 (2H, d, *J* = 8.9 Hz, aromatic-H), 13.26 (1H, s, NH). ^13^C-NMR (DMSO-*d*_6_, ppm) δ 41.3, 96.9 (d, *J* = 28.5 Hz), 105.0 (d, *J* = 23.3 Hz), 111.6 (d, *J* = 11.6 Hz), 124.2, 124.4, 127.5, 127.7, 128.8, 130.4, 132.1 (d, *J* = 10.4 Hz), 133.3, 140.1, 142.0, 150.8, 156.4 (d, *J* = 256.3 Hz), 163.9, 165.3, 192.6. HRMS (*m*/*z*): [M + H]^+^ calcd for C_23_H_14_BrFN_4_O_2_S: 509.0078; found: 509.0062.

*2-[(5-(4-(5(6)-Fluoro-1H-benzimidazol-2-yl)phenyl)-1,3,4-oxadiazol-2-yl)thio]-1-(4-nitro phenyl)ethan-1-one* (**4e**), Yield 67%, m.p. 250.9–254.6 °C. IR ν_max_ (cm^−1^): 3263 (N-H), 3026 (aromatic C-H), 2949 (aliphatic C-H), 1672 (C=O), 1585–1473 (C=N, C=C), 1294–1072 (C-N, C-O). ^1^H-NMR (DMSO-*d*_6_, ppm) δ 5.20 (2H, s, CO-CH_2_), 7.07–7.15 (1H, m, aromatic-H), 7.44 (1H, dd, *J*_1_ = 2.2 Hz, *J*_2_ = 9.4 Hz, aromatic-H), 7.62–7.66 (1H, m, aromatic-H), 7.82 (2H, d, *J* = 8.6 Hz, aromatic-H), 8.02 (2H, d, *J* = 8.6 Hz, aromatic-H), 8.13 (2H, d, *J* = 8.5 Hz, aromatic-H), 8.34 (2H, d, *J* = 8.5 Hz, aromatic-H), 13.27 (1H, s, NH). ^13^C-NMR (DMSO-*d*_6_, ppm) δ 41.0, 97.8 (d, *J* = 25.9 Hz), 106.2 (d, *J* = 25.9 Hz), 111.8 (d, *J* = 10.9 Hz), 124.3, 127.5, 127.7, 128.7, 130.0, 131.0, 131.3 (d, *J* = 10.4 Hz), 132.5, 133.2, 134.5, 141.3, 157.3 (d, *J* = 274.2 Hz), 164.0, 165.3, 192.5. HRMS (*m*/*z*): [M + H]^+^ calcd for C_23_H_14_FN_5_O_4_S: 476.0823; found: 476.0826.

*2-[(5-(4-(5(6)-Fluoro-1H-benzimidazol-2-yl)phenyl)-1,3,4-oxadiazol-2-yl)thio]-1-(4-cyanophenyl) ethan-1-one* (**4f**), Yield 64%, m.p. 243.8–247.7 °C. IR ν_max_ (cm^−1^): 3264 (N-H), 3093 (aromatic C-H), 2946 (aliphatic C-H), 2229 (C≡N), 1681 (C=O), 1608–1467 (C=N, C=C), 1305–1080 (C-N, C-O). ^1^H-NMR (DMSO-*d*_6_, ppm) δ 5.23 (2H, s, CO-CH_2_), 7.07–7.13 (1H, m, aromatic-H), 7.37–7.73 (2H, m, aromatic-H), 8.10 (4H, t, *J* = 8.7 Hz, aromatic-H), 8.23 (2H, d, *J* = 8.5 Hz, aromatic-H), 8.33 (2H, d, *J* = 8.5 Hz, aromatic-H), 13.26 (1H, s, NH). ^13^C-NMR (DMSO-*d*_6_, ppm) δ 41.1, 98.4 (d, *J* = 27.3 Hz), 105.0 (d, *J* = 21.8 Hz), 113.0 (d, *J* = 9.7 Hz), 116.3, 118.5, 124.2, 127.5, 127.7, 129.6, 130.1, 131.1 (d, *J* = 11.5 Hz), 133.3, 133.4, 138.7, 141.0, 157.6 (d, *J* = 229.9 Hz), 163.9, 165.3, 192.5. HRMS (*m*/*z*): [M + H]^+^ calcd for C_24_H_14_FN_5_O_2_S: 456.0925; found: 456.0918.

*2-[(5-(4-(5(6)-Fluoro-1H-benzimidazol-2-yl)phenyl)-1,3,4-oxadiazol-2-yl)thio]-1-(2,4-dichlorophenyl)ethan-1-one* (**4g**), Yield 72%, m.p. 254.7–256.7 °C. IR ν_max_ (cm^−1^): 3215 (N-H), 3086 (aromatic C-H), 2954 (aliphatic C-H), 1687 (C=O), 1585–1475 (C=N, C=C), 1303–1070 (C-N, C-O). ^1^H-NMR (DMSO-*d*_6_, ppm) δ 5.05 (2H, s, CO-CH_2_), 7.06–7.16 (1H, m, aromatic-H), 7.49–7.59 (1H, m, aromatic-H), 7.63–7.73 (2H, m, aromatic-H), 7.81 (1H, d, *J* = 2.0 Hz, aromatic-H), 7.96 (1H, d, *J* = 8.4 Hz, aromatic-H), 8.13 (2H, d, *J* = 8.4 Hz, aromatic-H), 8.35 (2H, d, *J* = 8.2 Hz, aromatic-H), 13.27 (1H, s, NH). ^13^C-NMR (DMSO-*d*_6_, ppm) δ 43.7, 98.9 (d, *J* = 24.9 Hz), 105.2 (d, *J* = 26.2 Hz), 111.7 (d, *J* = 10.5 Hz), 113.7, 125.0, 128.3, 128.4, 129.0, 130.5 (d, *J* = 13.8 Hz), 131.6, 132.8, 133.1, 134.1, 136.0, 138.5, 144.4, 157.2 (d, *J* = 261.7 Hz), 164.5, 166.1, 194.7. HRMS (*m*/*z*): [M + H]^+^ calcd for C_23_H_13_Cl_2_FN_4_O_2_S: 499.0193; found: 499.0170.

*2-[(5-(4-(5(6)-Fluoro-1H-benzimidazol-2-yl)phenyl)-1,3,4-oxadiazol-2-yl)thio]-1-(3,4-dihydroxyphenyl)-ethan-1-one* (**4h**), Yield 62%, m.p. 234.7–236.9 °C. IR ν_max_ (cm^−1^): 3234 (N-H, O-H), 3072 (aromatic C-H), 2927 (aliphatic C-H), 1660 (C=O), 1595–1467 (C=N, C=C), 1301–1080 (C-N, C-O). ^1^H-NMR (DMSO-*d*_6_, ppm) δ 5.08 (2H, s, CO-CH_2_), 6.88 (1H, d, *J* = 8.2 Hz, aromatic-H), 7.10 (1H, t, *J* = 8.9 Hz, aromatic-H), 7.43 (2H, s, aromatic-H), 7.51 (1H, d, *J* = 8.1 Hz, aromatic-H), 7.63 (1H, br s, aromatic-H), 8.11 (2H, d, *J* = 7.9 Hz, aromatic-H), 8.34 (2H, d, *J* = 7.9 Hz, aromatic-H), 13.26 (1H, s, NH). ^13^C-NMR (DMSO-*d*_6_, ppm) δ 40.9, 98.8 (d, *J* = 22.4 Hz), 103.9 (d, *J* = 24.9 Hz), 111.7 (d, *J* = 14.1 Hz), 115.6, 115.7, 122.7, 124.3, 127.2, 127.5, 127.7, 129.6, 130.6, 133.2, 144.6, 145.9, 152.2, 156.7 (d, *J* = 250.2 Hz), 164.4, 165.1, 191.1. HRMS (*m*/*z*): [M + H]^+^ calcd for C_23_H_15_FN_4_O_4_S: 463.0871; found: 463.0854.

*2-[(5-(4-(5(6)-Chloro-1H-benzimidazol-2-yl)phenyl)-1,3,4-oxadiazol-2-yl)thio]-1-phenylethan-1-one* (**4i**), Yield 74%, m.p. 157.2–159.1 °C. IR ν_max_ (cm^−1^): 3130 (N-H), 3084 (aromatic C-H), 2929 (aliphatic C-H), 1678 (C=O), 1579-1471 (C=N, C=C), 1294–1074 (C-N, C-O). ^1^H-NMR (DMSO-*d*_6_, ppm) δ 5.22 (2H, s, CO-CH_2_), 7.26 (1H, d, *J* = 7.4 Hz, aromatic-H), 7.61–7.73 (5H, m, aromatic-H), 8.11 (2H, t, *J* = 8.5 Hz, aromatic-H), 8.35 (2H, d, *J* = 7.6 Hz, aromatic-H), 13.34 (1H, s, NH). ^13^C-NMR (DMSO-*d*_6_, ppm) δ 41.1, 111.8, 113.5, 118.5, 120.0, 122.6, 124.5, 126.8, 127.5, 127.8, 129.0, 129.4, 133.1, 134.5, 140.0, 142.3, 160.1, 165.2, 193.2. HRMS (*m*/*z*): [M + H]^+^ calcd for C_23_H_15_ClN_4_O_2_S: 447.0677; found: 447.0665.

*2-((5-(4-(5(6)-Chloro-1H-benzo[d]imidazol-2-yl)phenyl)-1,3,4-oxadiazol-2-yl)thio)-1-(p-tolyl)ethan-1-one* (**4j**), Yield 68%, m.p. 201.5–203.7 °C. IR ν_max_ (cm^−1^): 3312 (N-H), 2938 (aliphatic C-H), 1674 (C=O), 1611–1474 (C=N, C=C), 1302–1024 (C-N, C-O). ^1^H-NMR (DMSO-*d*_6_, ppm) δ 2.41 (3H, s, CH_3_), 5.08 (2H, s, CH_2_), 7.23–7.29 (1H, m, aromatic-H), 7.40 (2H, d, *J* = 8.1 Hz, aromatic-H), 7.58 (1H, d, *J* = 8.6 Hz, aromatic-H), 7.70–7.76 (1H, m, aromatic-H), 7.98 (2H, d, *J* = 8.2 Hz, aromatic-H), 8.12 (2H, d, *J* = 8.5 Hz, aromatic-H), 8.35 (2H, d, *J* = 8.5 Hz, aromatic-H), 13.34 (1H, s, NH). ^13^C-NMR (DMSO-*d*_6_, ppm) δ 21.7, 41.1, 111.8, 116.4, 117.1, 120.9, 122.8, 124.5, 127.0, 127.5, 127.8, 129.1, 129.9, 133.0, 136.5, 140.6, 145.1, 162.1, 165.2, 192.6. HRMS (*m*/*z*): [M + H]^+^ calcd for C_24_H_17_ClN_4_O_2_S: 461.0834; found: 461.0818

*2-((5-(4-(5(6)-Chloro-1H-benzo[d]imidazol-2-yl)phenyl)-1,3,4-oxadiazol-2-yl)thio)-1-(4-chlorophenyl) ethan-1-one* (**4k**), Yield 78%, m.p. 229.4–231.2 °C. IR ν_max_ (cm^−1^): 3215 (N-H), 3086 (aromatic C-H), 2954 (aliphatic C-H), 1684 (C=O), 1583–1476 (C=N, C=C), 1304–1070 (C-N, C-O). ^1^H-NMR (DMSO-*d*_6_, ppm) δ 5.10 (2H, s, CH_2_), 7.23–7.29 (1H, m, aromatic-H), 7.43 (1H, br s, aromatic-H), 7.56–7.62 (1H, m, aromatic-H), 7.62 (2H, d, *J* = 8.2 Hz, aromatic-H), 7.90 (2H, d, *J* = 8.2 Hz, aromatic-H), 8.12 (2H, d, *J* = 8.5 Hz, aromatic-H), 8.35 (2H, d, *J* = 8.5 Hz, aromatic-H), 13.26 (1H, s, NH). ^13^C-NMR (DMSO-*d*_6_, ppm) δ 41.0, 123.0, 125.8, 126.9, 127.1, 127.5, 127.7, 127.8, 128.7, 129.4, 130.7, 131.3, 131.5, 132.2, 133.0, 152.4, 161.0, 165.0, 190.1. HRMS (*m*/*z*): [M + H]^+^ calcd for C_23_H_14_Cl_2_N_4_O_2_S: 481.0287; found: 481.0274.

*2-[(5-(4-(5(6)-Chloro-1H-benzimidazol-2-yl)phenyl)-1,3,4-oxadiazol-2-yl)thio]-1-(4-bromophenyl)ethan-1-one* (**4l**), Yield 65%, m.p. 205.3–207.6 °C. IR ν_max_ (cm^−1^): 3290 (N-H), 3034 (aromatic C-H), 2951 (aliphatic C-H), 1672 (C=O), 1585–1473 (C=N, C=C), 1303–1070 (C-N, C-O). ^1^H-NMR (DMSO-*d*_6_, ppm) δ 5.19 (2H, s, CO-CH_2_), 7.25 (1H, d, *J* = 8.5 Hz, aromatic-H), 7.57–7.72 (2H, m, aromatic-H), 7.82 (2H, d, *J* = 8.6 Hz, aromatic-H), 8.01 (2H, d, *J* = 8.6 Hz, aromatic-H), 8.12 (2H, d, *J* = 8.6 Hz, aromatic-H), 8.34 (2H, d, *J* = 8.6 Hz, aromatic-H), 13.32 (1H, s, NH). ^13^C-NMR (DMSO-*d*_6_, ppm) δ 41.0, 113.5, 121.5, 124.0, 124.4, 127.5, 127.8, 128.5, 128.7, 130.5, 131.0, 132.5, 133.1, 134.5, 139.0, 141.6, 164.0, 165.2, 192.5. HRMS (*m*/*z*): [M + H]^+^ calcd for C_23_H_14_BrClN_4_O_2_S: 524.9782; found: 524.9762.

*2-((5-(4-(5(6)-Chloro-1H-benzo[d]imidazol-2-yl)phenyl)-1,3,4-oxadiazol-2-yl)thio)-1-(4-nitrophenyl)ethan-1-one* (**4m**), Yield 68%, m.p. 211.2–213.8 °C. IR ν_max_ (cm^−1^): 3240 (N-H), 3062 (aromatic C-H), 2953 (aliphatic C-H), 1653 (C=O), 1525–1471 (C=N, C=C), 1344–1074 (C-N, C-O). ^1^H-NMR (DMSO-*d*_6_, ppm) δ 5.27 (2H, s, CH_2_), 7.23–7.29 (1H, m, aromatic-H), 7.58 (1H, d, *J* = 8.6 Hz, aromatic-H), 7.70–7.76 (1H, m, aromatic-H), 7.82 (2H, d, *J* = 8.2 Hz, aromatic-H), 7.99 (2H, d, *J* = 8.2 Hz, aromatic-H), 8.12 (2H, d, *J* = 8.5 Hz, aromatic-H), 8.35 (2H, d, *J* = 8.5 Hz, aromatic-H), 13.29 (1H, s, NH). ^13^C-NMR (DMSO-*d*_6_, ppm) δ 41.4, 112.5, 115.3, 120.1, 124.3, 124.4, 126.1, 127.5, 127.7, 127.8, 130.4, 131.3, 133.0, 140.1, 143.6, 150.7, 162.8, 165.3, 192.5. HRMS (*m*/*z*): [M + H]^+^ calcd for C_23_H_14_ClN_5_O_4_S: 492.0528; found: 492.0508.

*2-[(5-(4-(5(6)-Chloro-1H-benzimidazol-2-yl)phenyl)-1,3,4-oxadiazol-2-yl)thio]-1-(4-cyanophenyl) ethan-1-one* (**4n**), Yield 71%, m.p. 160.3–162.3 °C. IR ν_max_ (cm^−1^): 3246 (N-H), 3024 (aromatic C-H), 2949 (aliphatic C-H), 2242 (C≡N), 1681 (C=O), 1563–1471 (C=N, C=C), 1296–1055 (C-N, C-O). ^1^H-NMR (DMSO-*d*_6_, ppm) δ 5.24 (2H, s, CO-CH_2_), 7.26 (1H, d, *J* = 6.2 Hz, aromatic-H), 7.60–7.77 (2H, m, aromatic-H), 8.07–8.14 (4H, m, aromatic-H), 8.23 (2H, d, *J* = 8.6 Hz, aromatic-H), 8.35 (2H, d, *J* = 8.6 Hz, aromatic-H), 13.34 (1H, s, NH). ^13^C-NMR (DMSO-*d*_6_, ppm) δ 41.1, 116.3, 118.5, 118.9, 121.0, 123.0, 123.7, 124.4, 127.5, 127.8, 129.6, 133.1, 133.4, 138.7, 139.1, 142.8, 162.8, 163.9, 165.3, 192.8. HRMS (*m*/*z*): [M + H]^+^ calcd for C_24_H_14_ClN_5_O_2_S: 472.0629; found: 472.0608.

*2-[(5-(4-(5(6)-Chloro-1H-benzimidazol-2-yl)phenyl)-1,3,4-oxadiazol-2-yl)thio]-1-(2,4-dichlorophenyl)ethan-1-one* (**4o**), Yield 80%, m.p. 253.7–256.8 °C. IR ν_max_ (cm^−1^): 3215 (N-H), 3086 (aromatic C-H), 2954 (aliphatic C-H), 1683 (C=O), 1583–1475 (C=N, C=C), 1303–1070 (C-N, C-O). ^1^H-NMR (DMSO-*d*_6_, ppm) δ 5.05 (2H, s, CO-CH_2_), 7.26 (1H, br s, aromatic-H), 7.60–7.82 (4H, m, aromatic-H), 7.96 (1H, d, *J* = 8.3 Hz, aromatic-H), 8.13 (2H, d, *J* = 8.5 Hz, aromatic-H), 8.36 (2H, d, *J* = 8.6 Hz, aromatic-H), 13.35 (1H, s, NH). ^13^C-NMR (DMSO-*d*_6_, ppm) δ 42.9, 115.1, 119.1, 123.7, 124.4, 127.5, 127.8, 128.2, 128.8, 129.0, 130.8, 132.0, 132.2, 132.3, 133.1, 135.2, 137.7, 142.0, 163.8, 165.3, 194.0. HRMS (*m*/*z*): [M + H]^+^ calcd for C_23_H_13_Cl_3_N_4_O_2_S: 514.9898; found: 514.9881.

*2-[(5-(4-(5(6)-Chloro-1H-benzimidazol-2-yl)phenyl)-1,3,4-oxadiazol-2-yl)thio]-1-(3,4-dihydroxyphenyl)-ethan-1-one* (**4p**), Yield 68%, m.p. 152.4–155.8 °C. IR ν_max_ (cm^−1^): 3240 (N-H, O-H), 3062 (aromatic C-H), 2895 (aliphatic C-H), 1658 (C=O), 1595–1471 (C=N, C=C), 1300–1024 (C-N, C-O). ^1^H-NMR (DMSO-*d*_6_, ppm) δ 5.08 (2H, s, CO-CH_2_), 6.88 (1H, d, *J* = 8.2 Hz, aromatic-H), 7.26 (1H, dd, *J*_1_ = 8.6 Hz, *J*_2_ = 2.0 Hz aromatic-H), 7.43 (1H, d, *J* = 2.1 Hz, aromatic-H), 7.51 (1H, dd, *J*_1_ = 8.3 Hz, *J*_2_ = 2.2 Hz, aromatic-H), 7.62–7.68 (2H, m, aromatic-H), 8.12 (2H, d, *J* = 8.6 Hz, aromatic-H), 8.35 (2H, d, *J* = 8.6 Hz, aromatic-H), 13.22 (1H, s, N-H). ^13^C-NMR (DMSO-*d*_6_, ppm) δ 40.9, 115.6, 115.7, 122.4, 122.7, 123.3, 124.5, 127.2, 127.5, 127.8, 129.0, 130.2, 133.0, 138.5, 140.6, 145.9, 151.8, 152.2, 163.4, 165.1, 191.1. HRMS (*m*/*z*): [M + H]^+^ calcd for C_23_H_15_ClN_4_O_4_S: 479.0575; found: 479.0563.

### 3.3. Antifungal Assays

Compounds **4a**–**4p** were tested for their antifungal activity in concert with EUCAST definitive (EDef 7.1) method as reported in literature [53]. Amphotericin B was positive control. The in vitro growth inhibitory activity of the compounds were tested against *C. albicans* (ATCC 90030), *C. krusei* (ATCC 6258) and *C. parapsilopsis* (ATCC 22019). The results were obtained as MIC values and MIC readings were accomplished twice for each chemical agent.

The fungal strains were incubated at 37 °C overnight, then sustained in Roswell Park Memorial Institute (RPMI) medium. Inoculum density was adjusted to 0.5 McFarland turbidity standard by McFarland Densitometer (DEN-1B Mcfarland densitometer, Biosan, Riga, Latvia). The resulting inoculum suspension contains 0.5–2.5 × 10^5^ cfu/mL for fungi. DMSO was used as solvent. Resazurin (20 µg/mL) was used to observe the fungal growth. MIC_50_ values were determined with a microplate reader at 590 nm excitation, 560 nm emission. Each experiment in antifungal assay was performed twice. The details of the anticandidal assay were reported in our previous study [55].

### 3.4. Cytotoxicity Assay

The cytotoxicity of the most active compounds (**4h** and **4p**) was determined by MTT assay as previously reported [56]. NIH/3T3 mouse embryonic fibroblast cells (ATCC^®^ CRL-1658™, London, UK) were incubated according to the supplier’s recommendations and seeded as 1 × 10^4^ cells into each well of 96-well plates. The IC_50_ values were determined by plotting a dose-response curve of inhibition % versus tested concentrations of the compound.

### 3.5. Quantification of Ergosterol Level

Ergosterol level was determined using the extract of total sterols from *C. albicans* following the method described by Breivik and Owades [57] with arrangements as described in our previous work [51]. In order to calculate IC_50_ values against ergosterol biosynthesis, compounds and reference agent were tested at five different concentrations (0.49 µg/mL, 0.98 µg/mL, 1.95 µg/mL, 3.91 µg/mL and 7.81 µg/mL).

### 3.6. Molecular Docking Studies

Molecular docking studies were performed by using in silico procedure to define the binding modes of compounds **4h** and **4p** on 14 α-sterol demethylase enzyme active region. X-ray crystal structures of 14 α-sterol demethylase enzyme (PDB code: 5FSA) [54] were retrieved from the Protein Data Bank server (www.pdb.org). The docking procedure was applied using Schrödinger Maestro [58] interface as in previously described by our research group [8,52,59,60].

## 4. Conclusions

As part of our ongoing efforts to develop new antifungal heterocyclic compounds, in the current study, new benzimidazole-oxadiazole compounds **4a**–**4p** were synthesized by a four-step methodology and evaluated for their anticandidal activity. Among all the compounds **4h** and **4p** demonstrated comparable inhibitory activity on the growth of tested *Candida* species as positive controls and were selected as the lead compounds. Two hydroxy groups at *m*- and *p*-positions (compounds **4h** and **4p**) potentiated the biological activity. MTT assays revealed that these compounds are nontoxic against three tested strains at their active concentrations. Inhibition of ergosterol biosynthesis on fungal membranes may be their mechanism of antifungal action. These findings suggest that **4h** and **4p** are promising lead compounds for the enhancement of new agents in the therapy of fungal infections. In conclusion, we believe that the obtained results add value to our study program in this field and will guide our future studies.

## Figures and Tables

**Table 1:** Some characteristics and MIC_50_ (μg/mL) values of compounds (**4a**–**4s**).

Compound	R_1_	R_2_	R_3_	R_4_	*C. albicans*	*C. krusei*	*C. parapsilopsis*
**4a**	F	H	H	H	>1000	>1000	>1000
**4b**	F	H	H	CH_3_	>1000	>1000	>1000
**4c**	F	H	H	Cl	1000	1000	1000
**4d**	F	H	H	Br	500	1000	>1000
**4e**	F	H	H	NO_2_	>1000	>1000	>1000
**4f**	F	H	H	CN	>1000	>1000	>1000
**4g**	F	Cl	H	Cl	>1000	>1000	>1000
**4h**	F	H	OH	OH	1.95	31.25	62.5
**4i**	Cl	H	H	H	500	>1000	>1000
**4j**	Cl	H	H	CH_3_	>1000	>1000	>1000
**4k**	Cl	H	H	Cl	>1000	>1000	>1000
**4l**	Cl	H	H	Br	1000	>1000	>1000
**4m**	Cl	H	H	NO_2_	1000	>1000	>1000
**4n**	Cl	H	H	CN	>1000	>1000	>1000
**4o**	Cl	Cl	H	Cl	>1000	>1000	>1000
**4p**	Cl	H	OH	OH	1.95	7.8	31.25
Amphotericin B	-	-	-	-	1.95	1.95	1.95
Ketoconazole	-	-	-	-	7.8	1.95	1.95

**Table 2:** IC_50_ of the compounds **4h**, **4p** and ketoconazole on ergosterol biosynthesis of *C. albicans*.

Compound	IC_50_ (µg/mL)
**4h**	2.84 ± 0.16
**4p**	3.28 ± 0.22
Ketoconazole	0.66 ± 0.07

## Supplementary Materials

The following are available online, Figures S1–S16: ^1^H-NMR spectra of compounds **4a**–**4p**.
