# Supplementary material for: Synthesis and Antifungal Potential of Some Novel Benzimidazole-1,3,4-Oxadiazole Compounds
Source: Molecules. 2019 Jan 6;24(1):191. doi: 10.3390/molecules24010191 (PMC6337182; doi:10.3390/molecules24010191)
Supplement: Supplementary file 1 [file molecules-24-00191-s001.pdf]

Supplementary Material

Synthesis and Antifungal Potential of Some Novel Benzimidazole-1,3,4-Oxadiazole Compounds

Ahmet Çağrı Karaburun [1], Betül Kaya Çavuşoğlu [1], Ulviye Acar Çevik [1,2], Derya Osmaniye [1,2], Begüm Nurpelin Sağlık [1,2], Serkan Levent [1,2], Yusuf Özkay [1,2], Özlem Atlı [3], Ali Savaş Koparal [4] and Zafer Asım Kaplancıklı [1,*]

1 Department of Pharmaceutical Chemistry, Faculty of Pharmacy, Anadolu University, Eskişehir 26470, Turkey; ackarabu@anadolu.edu.tr (A.Ç.K.); betulkaya@anadolu.edu.tr (B.K.Ç.); uacar@anadolu.edu.tr (U.A.Ç.); dosmaniye@anadolu.edu.tr (D.O.); bnsaglik@anadolu.edu.tr (B.N.S.); serkanlevent@anadolu.edu.tr (S.L.); yozkay@anadolu.edu.tr (Y.Ö.)

2 Doping and Narcotic Compounds Analysis Laboratory, Faculty of Pharmacy, Anadolu University, Eskişehir 26470, Turkey

3 Department of Pharmaceutical Toxicology, Faculty of Pharmacy, Anadolu University, Eskişehir 26470, Turkey; oatli@anadolu.edu.tr

4 Open Education Faculty, Anadolu University, Eskişehir 26470, Turkey; askopara@anadolu.edu.tr

* Correspondence: zakaplan@anadolu.edu.tr; Tel.: +90-222-335-0580/3775

Academic Editor: Fawaz Aldabbagh

Received: 5 December 2018; Accepted: 30 December 2018; Published: date

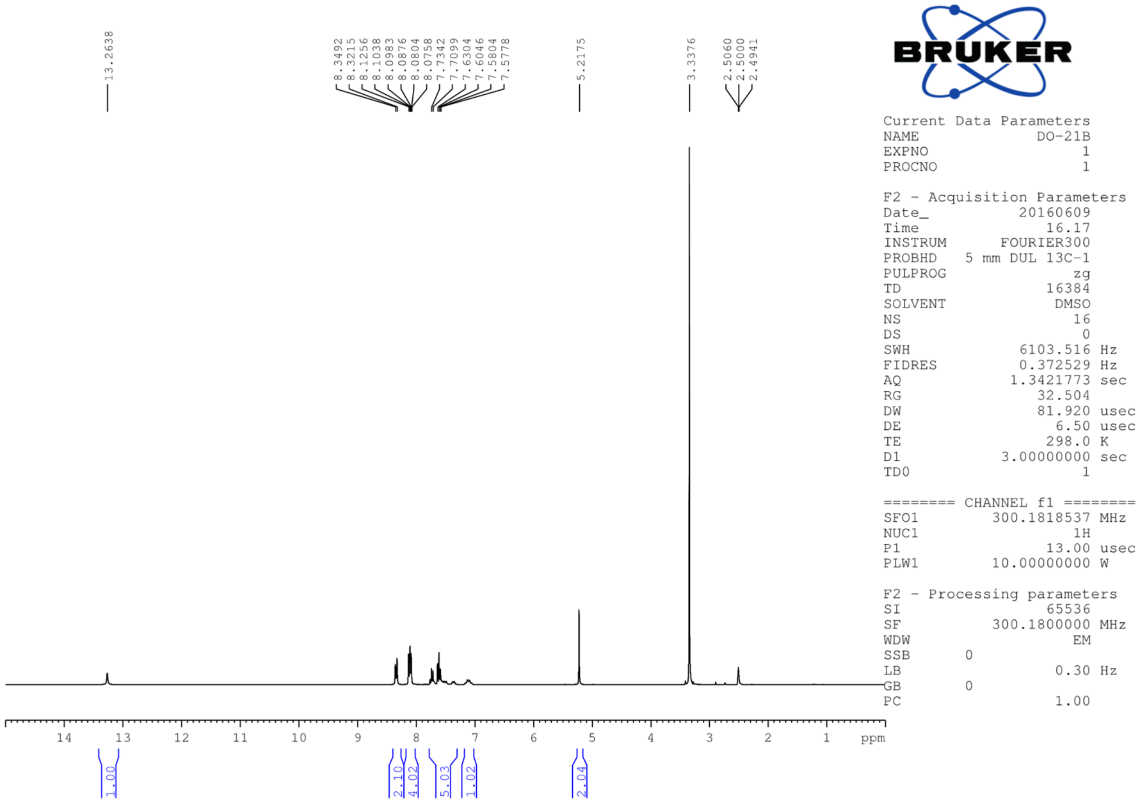

Figure S1. ^{1}H-NMR spectrum of compound **4a**

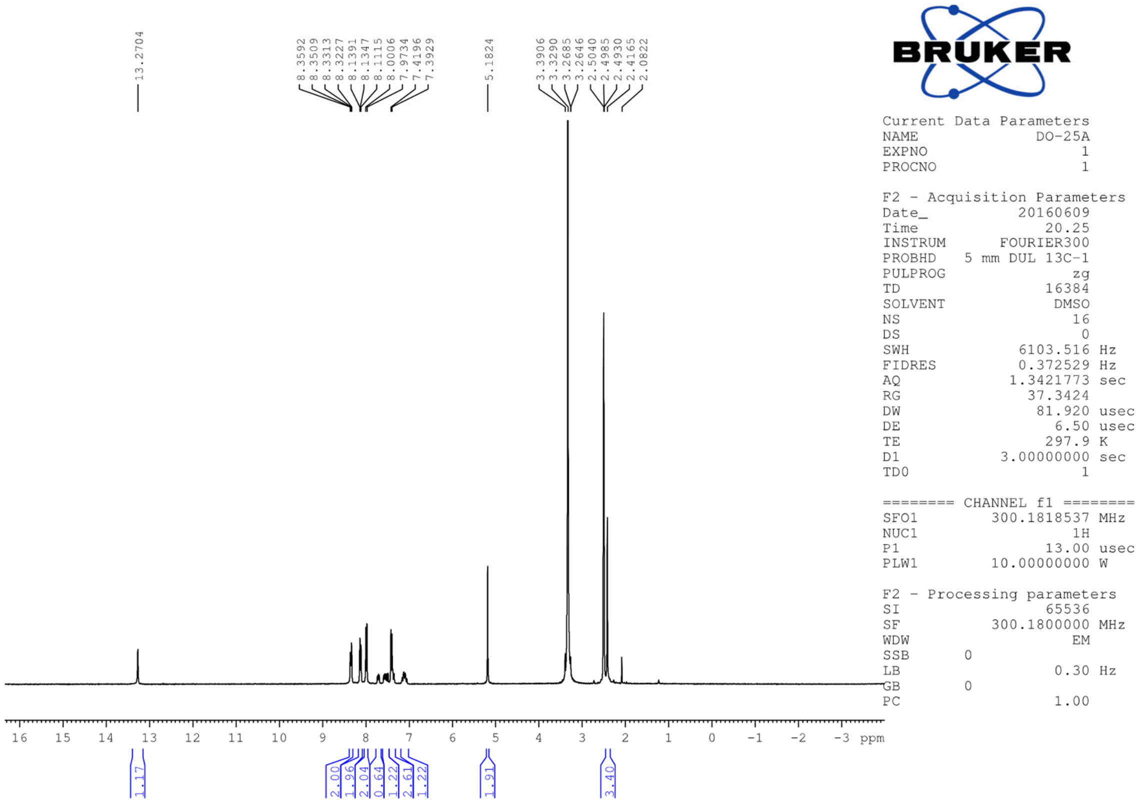

Figure S2. ^{1}H-NMR spectrum of compound **4b**

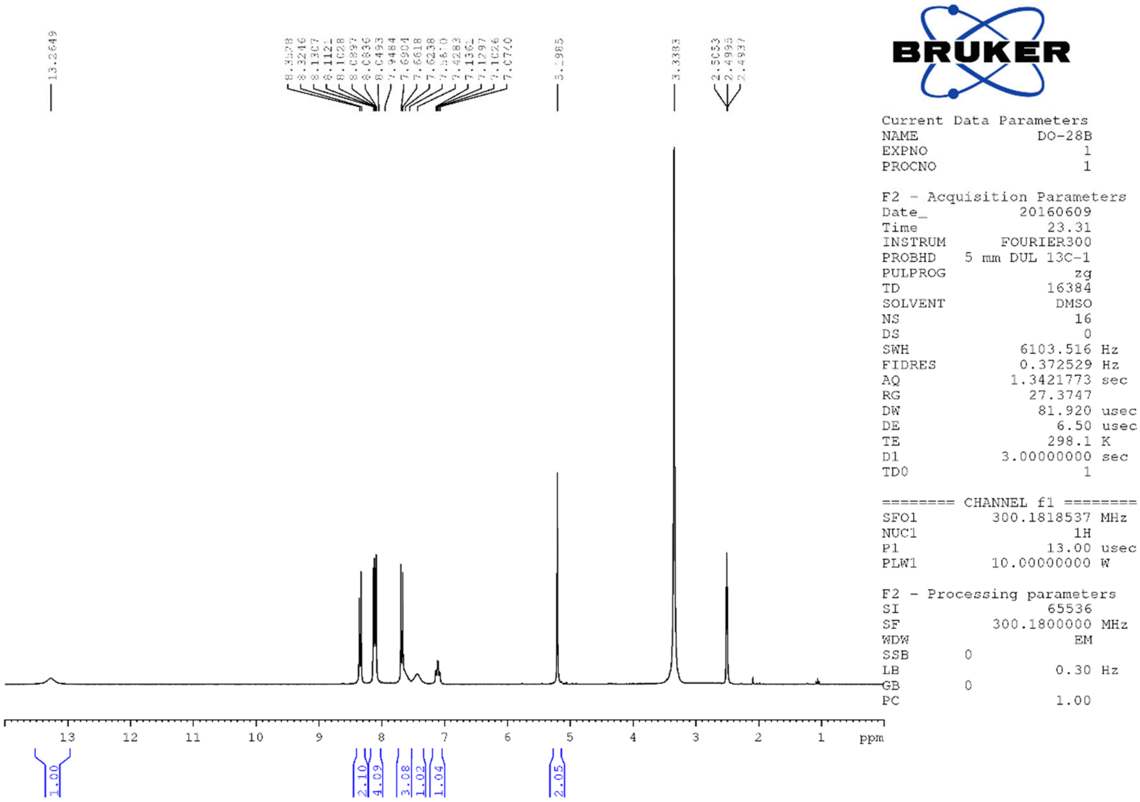

Figure S3. ^{1}H-NMR spectrum of compound **4c**

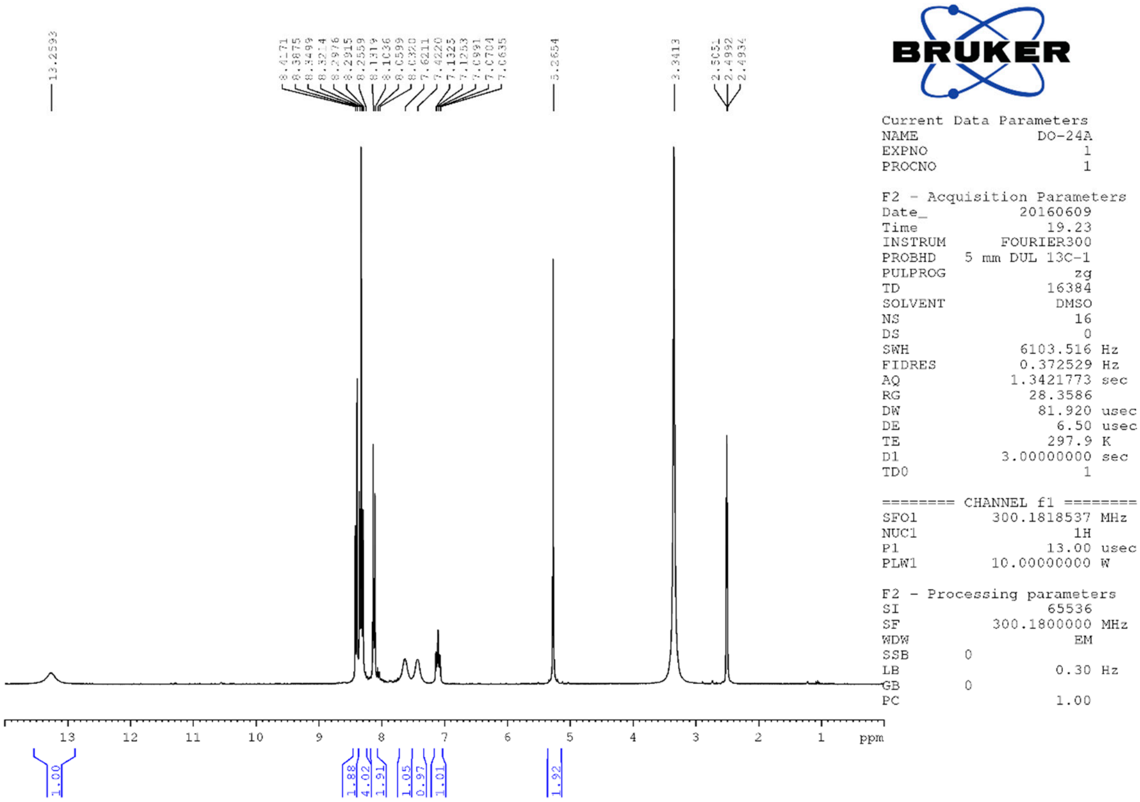

Figure S4. ^{1}H-NMR spectrum of compound **4d**

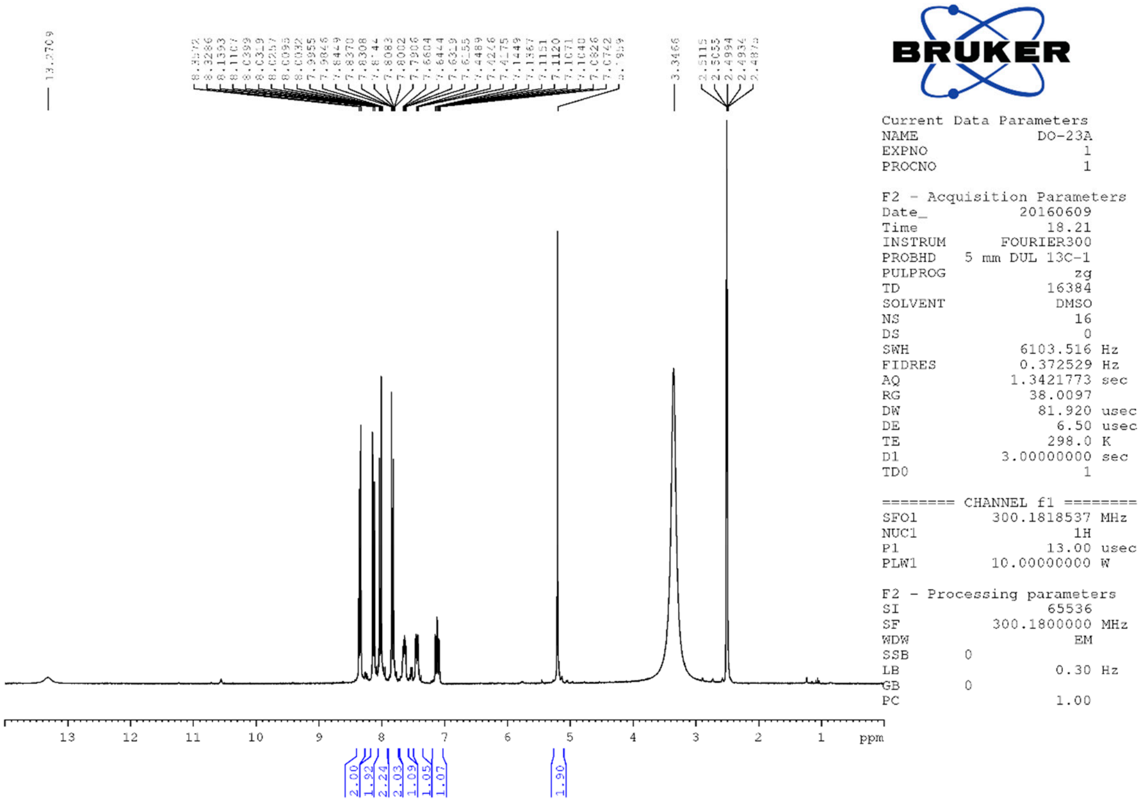

Figure S5. ^{1}H-NMR spectrum of compound **4e**

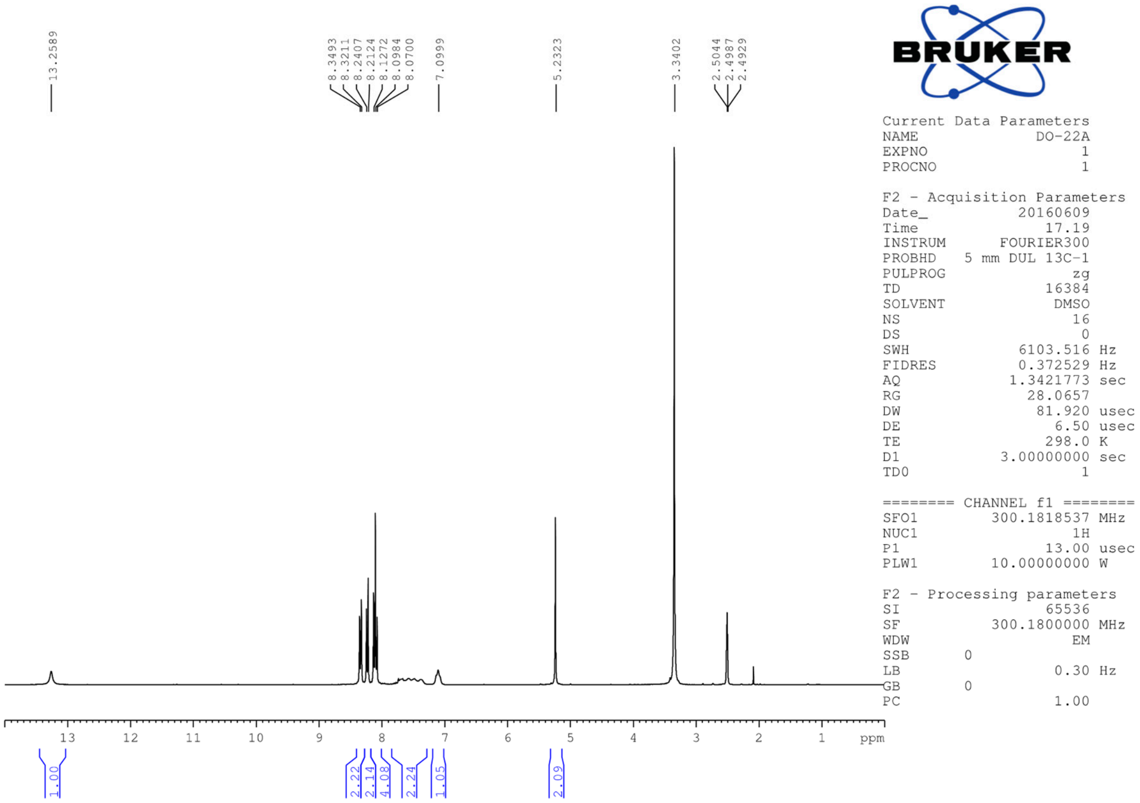

Figure S6. ^{1}H-NMR spectrum of compound **4f**

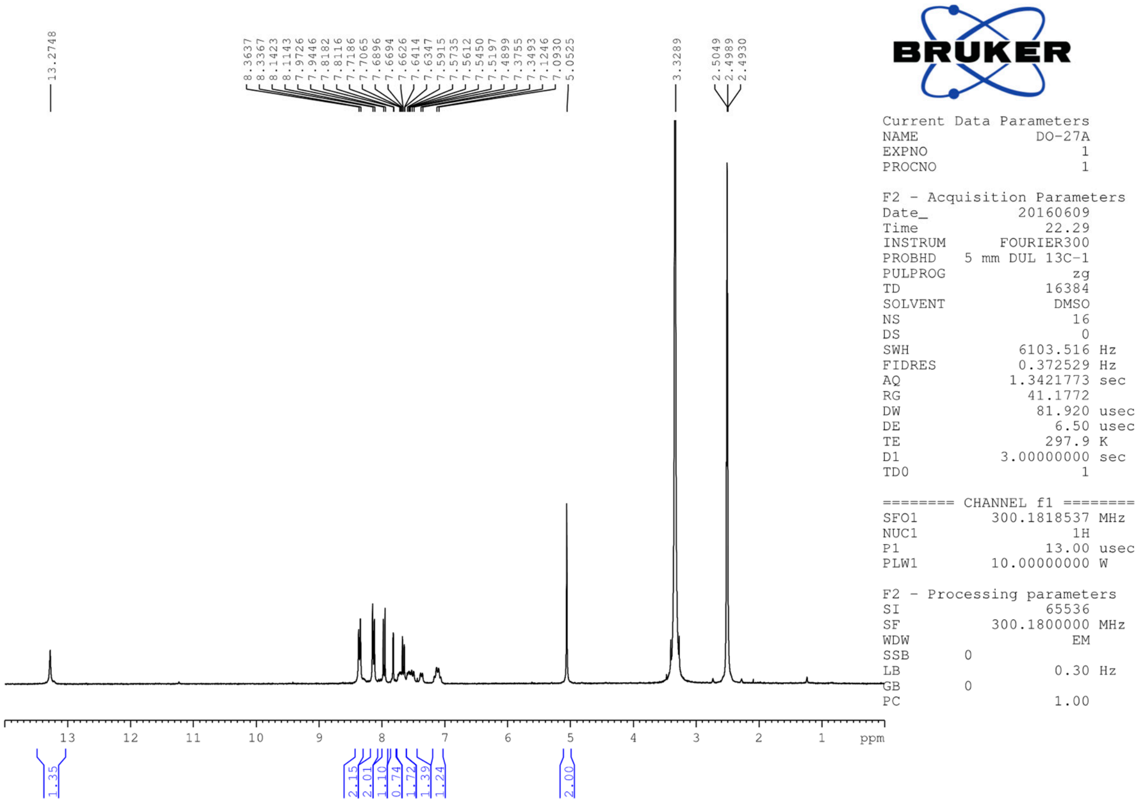

Figure S7. ^{1}H-NMR spectrum of compound **4g**

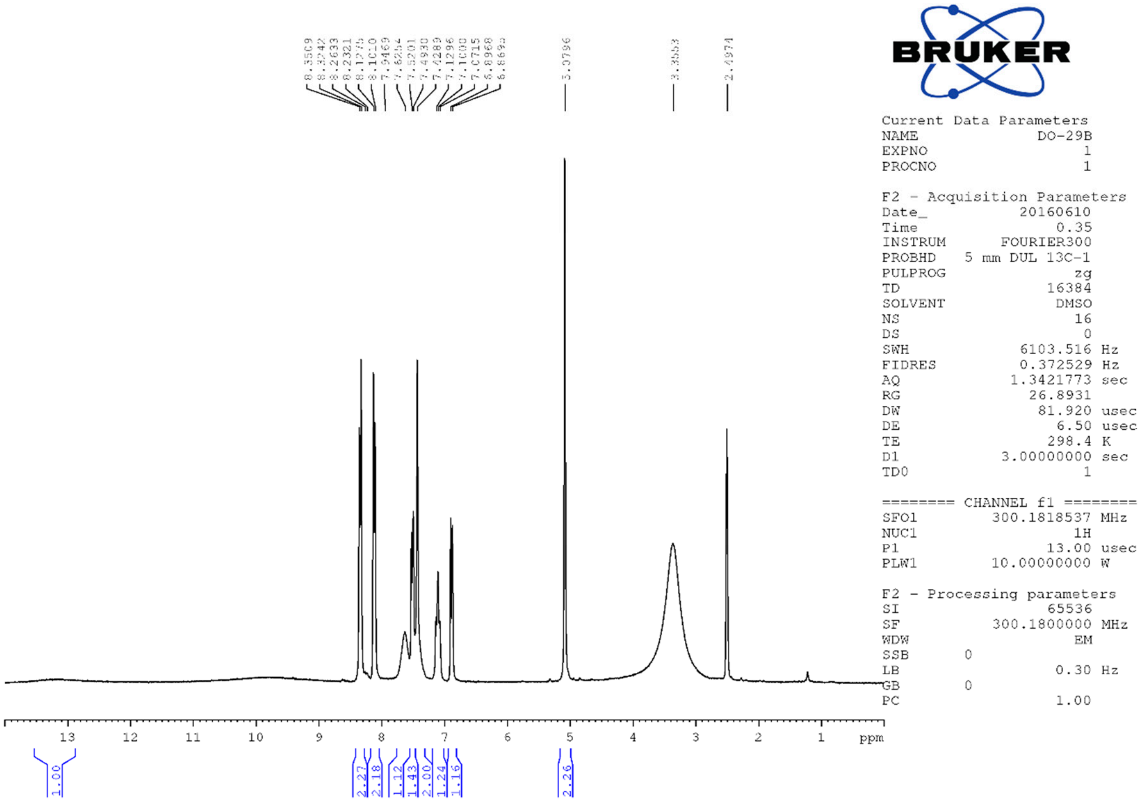

Figure S8. ^{1}H-NMR spectrum of compound **4h**

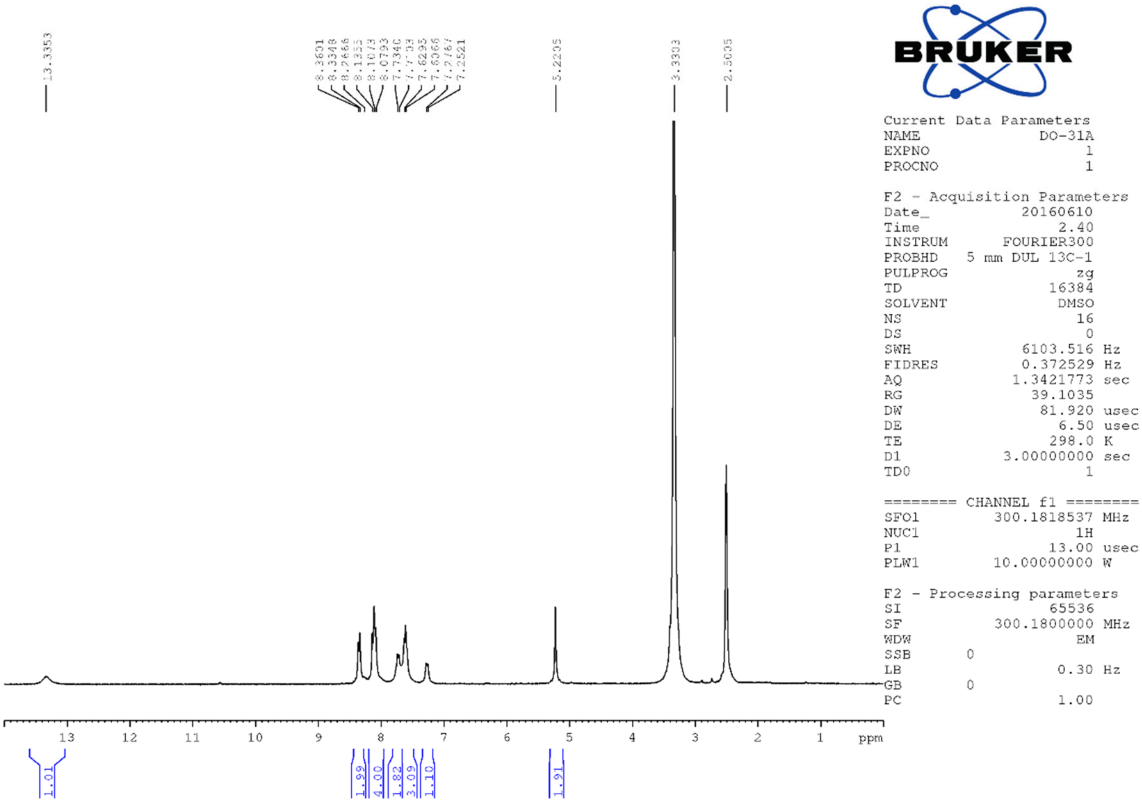

Figure S9. ^{1}H-NMR spectrum of compound **4i**

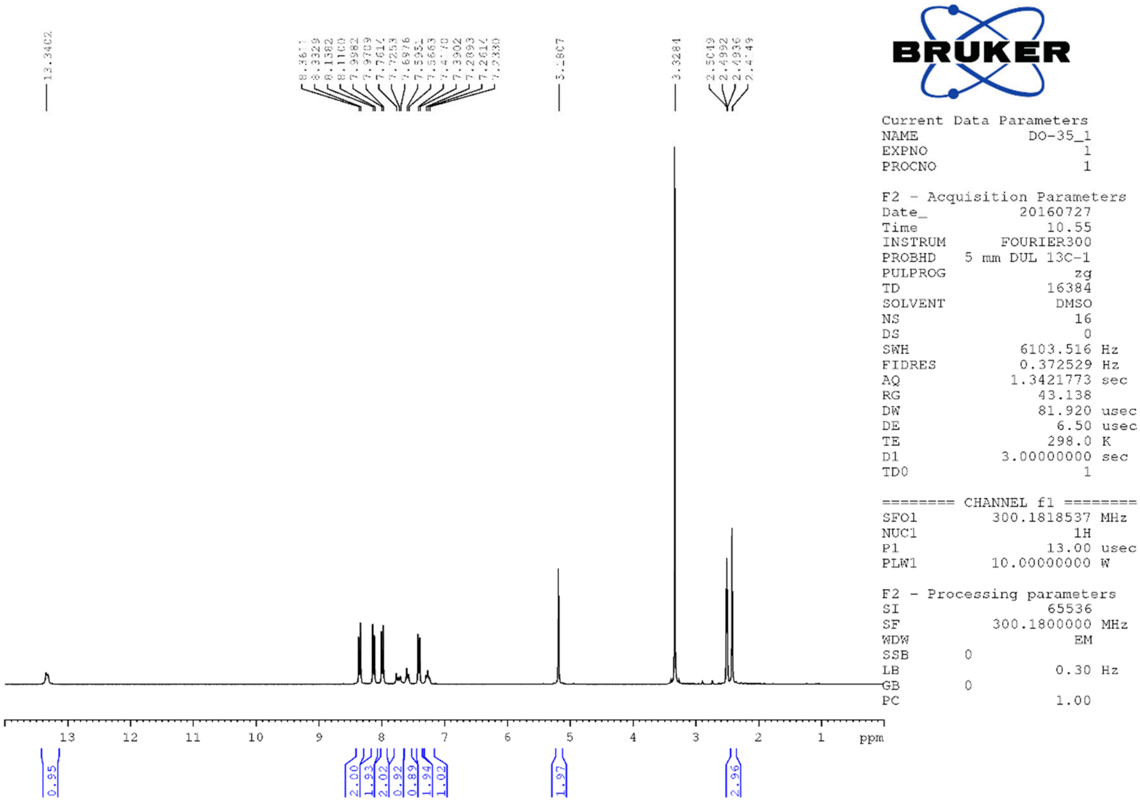

Figure S10. ^{1}H-NMR spectrum of compound **4j**

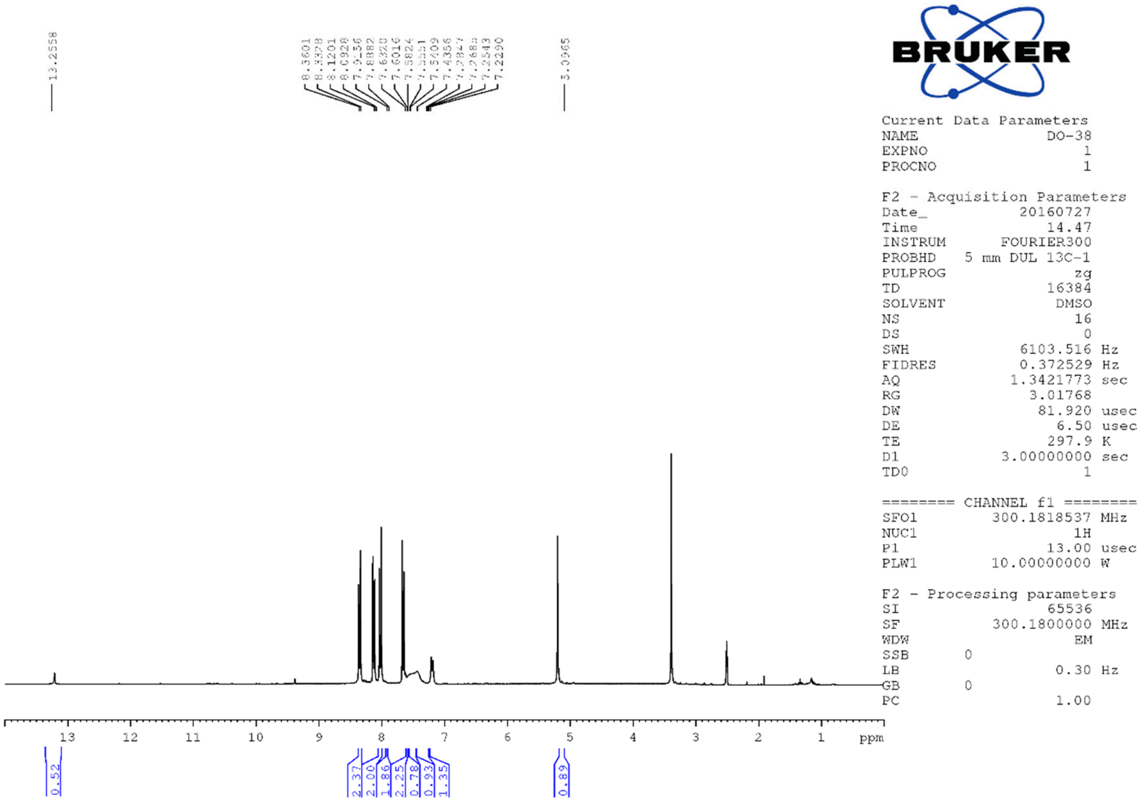

Figure S11. ^{1}H-NMR spectrum of compound **4k**

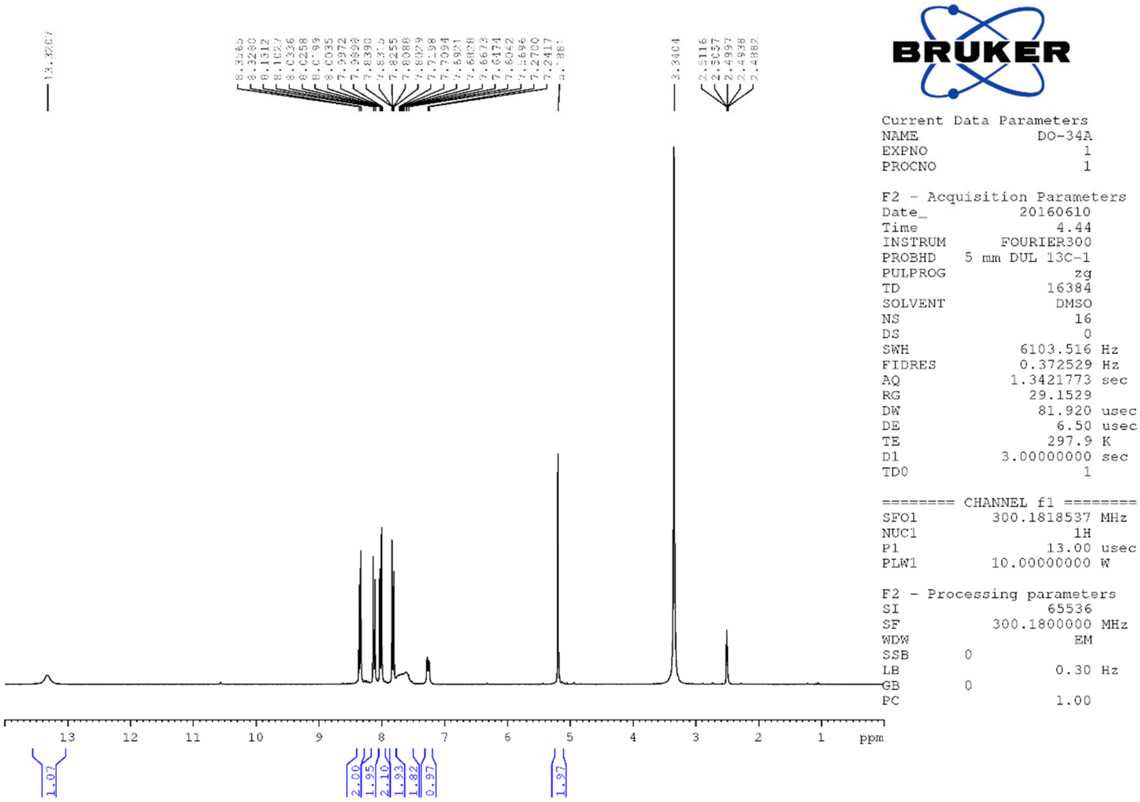

Figure S12. ^{1}H-NMR spectrum of compound **4l**

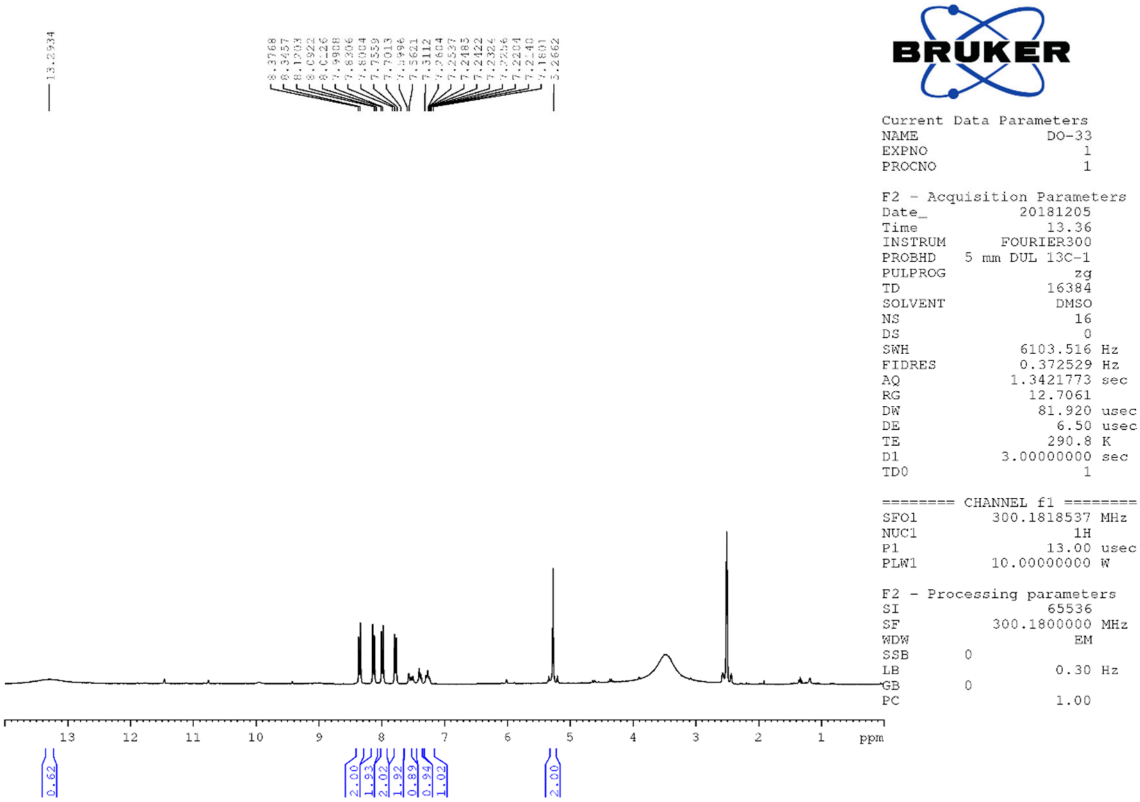

Figure S13. ^{1}H-NMR spectrum of compound **4m**

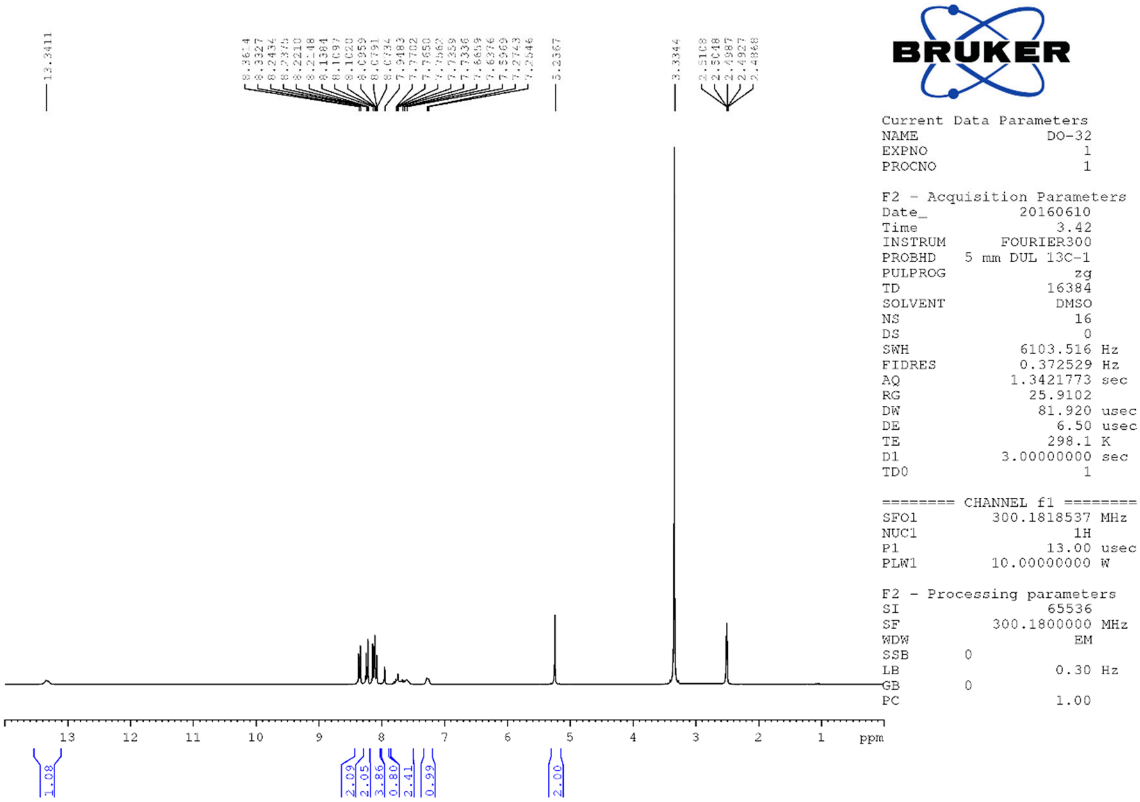

Figure S14. ^{1}H-NMR spectrum of compound **4n**

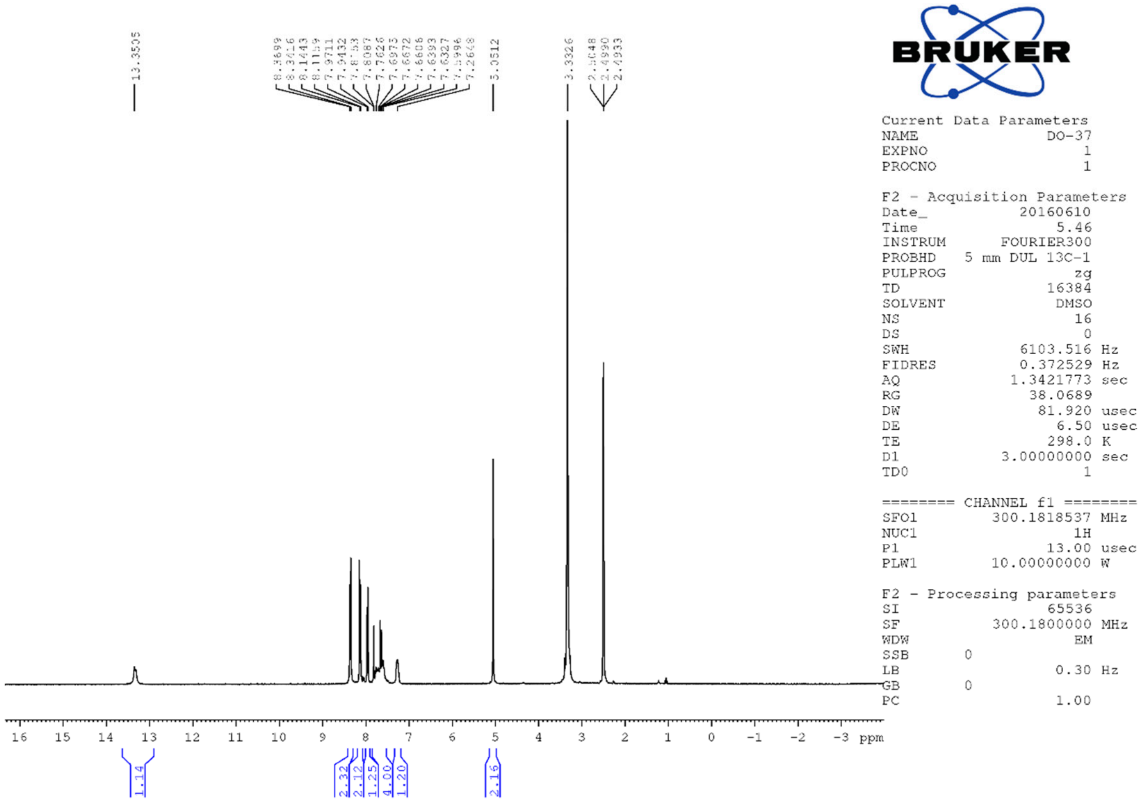

Figure S15. ^{1}H-NMR spectrum of compound **4o**

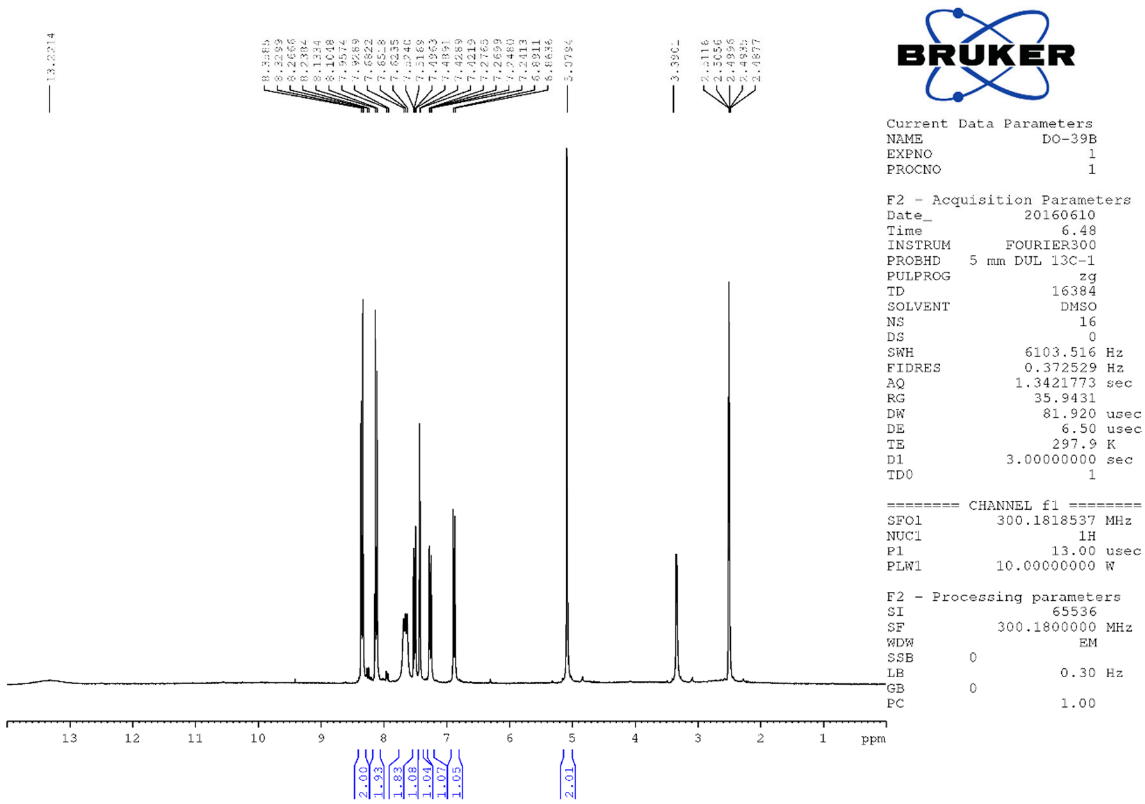

Figure S16. ^{1}H-NMR spectrum of compound **4p**
